# Potentially harmful excipients in neonatal medications: a multicenter nationwide observational study in Japan

**DOI:** 10.1186/s40780-021-00208-9

**Published:** 2021-07-01

**Authors:** Jumpei Saito, Naomi Nadatani, Makoto Setoguchi, Masahiko Nakao, Hitomi Kimura, Mayuri Sameshima, Keiko Kobayashi, Hiroaki Matsumoto, Naoki Yoshikawa, Toshihiro Yokoyama, Hitomi Takahashi, Mei Suenaga, Ran Watanabe, Kinuko Imai, Mami Obara, Mari Hashimoto, Kazuhiro Yamamoto, Naoko Fujiwara, Wakako Sakata, Hiroaki Nagai, Takeshi Enokihara, Sayaka Katayama, Yuta Takahashi, Mariko Araki, Kanako Iino, Naoko Akiyama, Hiroki Katsu, Kumiko Fushimi, Tomoya Takeda, Mayumi Torimoto, Rina Kishi, Naoki Mitsuya, Rie Kihara, Yuki Hasegawa, Yukihiro Hamada, Toshimi Kimura, Masaki Wada, Ayano Tanzawa, Akimasa Yamatani

**Affiliations:** 1 Department of Pharmacy, National Center for Child Health and Development, 2-10-1 Okura, Setagaya-ku, Tokyo, 175-8535 Japan; 2 Department of Pharmacy, Saitama Medical Center, Kawagoe-shi, Saitama, Japan; 3 Department of Pharmacy, Kagoshima City Hospital, Kagoshima-shi, Kagoshima, Japan; 4 Department of Clinical Research Center, Osaka City General Hospital, Osaka-shi, Osaka, Japan; 5 Department of Pharmacy, Osaka City General Hospital, Osaka-shi, Osaka, Japan; 6 Department of Pharmacy, Nagano Children’s Hospital, Azumino-shi, Nagano, Japan; 7 Division of Pharmacy, Ohara HealthCare Foundation Kurashiki Central Hospital, Kurashiki-shi, Okayama, Japan; 8 Department of Pharmacy, University of Miyazaki Hospital, Miyazaki-shi, Miyazaki, Japan; 9 Department of Pharmacy, Japanese Red Cross Nagoya Daiichi Hospital, Nagoya-shi, Aichi, Japan; 10 Department of Pharmacy, Anjo Kosei Hospital, Anjo-shi, Aichi, Japan; 11 Department of Pharmacy, National Hospital Organization Saga National Hospital, Saga-shi, Saga, Japan; 12 Department of Pharmacy, Teikyo University Hospital, Itabashi-ku, Tokyo, Japan; 13 Department of Pharmacy, Iwate Medical University, Morioka-shi, Iwate, Japan; 14 Department of Pharmacy, Kobe University Hospital, Kobe-shi, Hyogo, Japan; 15 Department of Pharmacy, Nihon University Itabashi Hospital, Itabashi-ku, Tokyo, Japan; 16 Department of Pharmacy, Hyogo Prefectural, Amagasaki General Medical Center, Amagasaki-shi, Hyogo, Japan; 17 Department of Pharmacy, Dokkyo Medical University Hospital, Mibu-shi, Tochigi, Japan; 18 Department of Pharmacy, Saitama Children’s Medical Center, Saitama-shi, Saitama, Japan; 19 Division of Pharmacy, Niigata University Medical and Dental Hospital, Niigata-shi, Niigata, Japan; 20 Department of Pharmacy, Osaka Women’s and Children’s Hospital, Izumi-shi, Osaka, Japan; 21 Department of Pharmacy, Hospital of the University of Occupational and Environmental Health, Kitakyushu-shi, Fukuoka, Japan; 22 Department of Pharmacy, National Hospital Organization Mie Chuo Medical Center, Tsu-shi, Mie, Japan; 23 Department of Pharmacy, Japanese Red Cross Kyoto Daiichi Hospital, Kyoto-shi, Kyoto, Japan; 24 Department of Hospital Pharmacy, Nagoya University Hospital, Nagoya-shi, Aichi, Japan; 25 Department of Pharmacy, Japanese Red Cross Otsu Hospital, Otsu-shi, Shiga, Japan; 26 Department of Pharmacy, National Hospital Organization Kyushu Medical Center, Fukuoka-shi, Fukuoka, Japan; 27 Department of Pharmacy, Tokyo Women’s Medical University Hospital, Shinjuku-ku, Tokyo, Japan; 28 Department of Neonatology, Maternal and Perinatal Center, Tokyo Women’s Medical University, Shinjuku-ku, Tokyo, Japan

**Keywords:** Excipient, Neonate, Enteral, Parenteral, Topical, Comparative study

## Abstract

**Background:**

A multicenter investigation of neonate exposure to potentially harmful excipients (PHEs) in neonatal intensive care units (NICUs) in Japan has not been conducted.

**Methods:**

A multicenter nationwide observational study was conducted. Neonate patient demographic data and information on all medicines prescribed and administered during hospitalization on 1 day between November 2019 and March 2021 were extracted from the medical records. Nine PHEs, paraben, polysorbate 80, propylene glycol, benzoates, saccharin sodium, sorbitol, ethanol, benzalkonium chloride, and aspartame, were selected. PHEs were identified from the package insert and the Interview Form. The quantitative daily exposure was calculated if quantitative data were available for each product containing the PHE.

**Results:**

Prescription data was collected from 22 NICUs in Japan. In total, 343 neonates received 2360 prescriptions for 426 products containing 228 active pharmaceutical ingredients. PHEs were found in 52 (12.2%) products in 646 (27.4%) prescriptions for 282 (82.2%) neonates. Benzyl alcohol, sodium benzoates, and parabens were the most common PHEs in parenteral, enteral, and topical formulations, respectively. Quantitative analysis showed that 10 (10%), 38 (42.2%), 37 (94.9%), and 9 (39.1%) neonates received doses exceeding the acceptable daily intake of benzyl alcohol, polysorbate 80, propylene glycol, and sorbitol, respectively. However, due to the lack of quantitative information for all enteral and topical products, accurate daily PHE exposure could not be quantified.

**Conclusions:**

Neonates admitted to NICUs in Japan were exposed to PHEs, and several of the most commonly prescribed medicines in daily clinical practice in NICUs contained PHEs. Neonate PHE exposure could be reduced by replacing these medicines with available PHE-free alternatives.

## Introduction

Excipients have many functions in pharmaceuticals, including solubility and stability modulation, and as preservative agents, colorants, or sweeteners, which are necessary to maintain quality and improve patient acceptability of medicines [1]. Ideally, excipients have limited pharmacological activity; however, some are associated with toxicity in neonates [2–6]. Excipient pharmacokinetics in neonates differ from adults and older children and may also be affected by underlying diseases [1]. Thus, consideration of excipients is essential when developing age-appropriate neonatal medicine formulations [2]. Conducting high-quality risk assessments and setting specific limits for excipients in pharmaceutical products for neonates will facilitate the prevention of adverse side-effects in clinical settings and regulatory mistakes [4].

Several reports suggest that potentially harmful excipients (PHEs) are included in drugs prescribed for neonates [4, 7–13]. The safety of PHEs, such as benzyl alcohol, ethanol, parabens, and propylene glycol, for pediatric patients, including neonates, has been investigated [14–16]. Commercially available pediatric dosage forms in Japan are different from other countries. In Japan, solid dosage forms are mainly administered as oral dosage forms, which reduces the amounts of sweeteners, dissolving agents, and preservative agents required compared to liquid dosage forms; thus, the frequency of neonatal exposure to excipients may be assumed to be different [17–19]. A single-center observational study was previously conducted in Japan [20]; however, a nationwide investigation of excipient use in neonatal medications has not been reported.

In this study, we conducted a nationwide multicenter investigation to determine the extent of PHE administration in neonatal intensive care units (NICU) in Japan.

## Methods

A multicenter point-prevalence study was conducted. NICUs in perinatal medical centers in Japan with more than nine beds for neonatal intensive care were invited to record prescriptions. Data were collected for 1 day (24 h) chosen by the unit between 1 November 2019 and 30 March 2021. Demographic data and all prescriptions were extracted from the medical charts in each hospital and registered. Each medicine was classified according to trade name (product), manufacturer, pharmaceutical dosage form, strength, and administration route (even the product contained the same active pharmaceutical ingredient, the products made by other manufacturer was regarded as different product).

### Identification of excipients

Nine excipients were selected as PHEs: aspartame (L-aspartyl L-phenylalanine methyl ester), benzalkonium chloride, benzoates (benzoic acid, sodium benzoate, and benzyl alcohol), ethanol, parabens (ethyl-, methyl-, and propyl-parabens), polysorbate 80, propylene glycol, saccharin sodium, and sorbitol. All PHEs, except aspartame, were described and studied previously [20, 21]. Aspartame, which is an artificial sugar substitute used as a sweetener to improve the palatability of pharmaceuticals, was included as a PHE in this study because the safety of aspartame for neonates has been questioned but not yet determined [22–26]. The PHE content of each medicine was identified from package inserts.

### Safety assessment

The daily exposure to PHEs (mg/kg/day) was calculated based on available data on the quantities of excipients in each product. The safety of daily exposures was assessed based on the previously reported acceptable daily intake (ADI) of aspartame [24, 25, 27], benzalkonium chloride [28], benzyl alcohol [29, 30], benzoates [29], ethanol [15], parabens [31], polysorbate 80 [32], propylene glycol [33, 34], saccharin sodium [27], and sorbitol [35] for pediatric or adult populations. The daily PHE dose (mg/kg body weight) was calculated only if the quantitative information was available. The product, neonate, and prescription numbers for missing quantitative data were counted.

## Results

### Demographics and prescriptions

Of 353 NICUs (3289 beds) in Japan, all 108 general perinatal medical centers were invited to participate in our survey. Of them, 38 (10.8%) of facilities responded and 22 perinatal medical facilities (6.2%) sent us the survey data after approval by the ethics committee in each hospital. We collected data from 343 neonate cases, accounting for 0.49% of the total number of NICU admissions in 2017. The median gestational age was 31.9 (21.4–41.4) weeks. The median postnatal age and postmenstrual age (PMA) at the time of the investigation was 17 (0–267) days and 36.0 (23.3–71.1) weeks, respectively. The median birth weight and body weight at the time of the investigation was 1470 (280–4030) g and 1972 (410–7505) g, respectively.

In total, 343 neonates received 2360 prescriptions for 426 products containing 228 active pharmaceutical ingredients (API). Among them, 225 neonates received 1475 prescriptions for 291 parenteral medicine products containing 114 APIs; 200 neonates received 763 prescriptions for 104 enteral medicine products containing 88 APIs; 82 neonates received 122 prescriptions for 31 topical products containing 26 APIs. The patients’ demographic characteristics (gestational age, postnatal age, birth weight, and body weight at the time of the investigation) and prescription data are shown in Table 1.

**Table 1:** Patient demographics and prescription status

**Patients information**			
Facilities	22		
Case	343		
Gestational age (weeks), median (range)	31.9 (21.4–41.4)		
Postnatal age (days), median (range)	17 (0–267)		
Birth weight (g), median (range)	1470 (280–4030)		
Bodyweight at dose (g), median (range)	1972 (410–7505)		
PMA (weeks), median (range)	36.0 (23.3–71.1)		
**Prescription information**	**No. (%) of neonates**	**No. (%) of prescriptions**	**No. (%) of products**
Total	343	2360	426
Parenteral	225 (65.6%)	1475 (62.5%)	291 (68.3%)
Enteral	200 (58.3%)	763 (32.3%)	104 (24.4%)
Topical	82 (23.9%)	122 (5.2%)	31 (7.3%)

### PHE administration frequency

Excipient information was available for 426 (100%) products in 2360 (100%) prescriptions. PHEs were identified in 52 (12.2%) products in 646 (23.4%) prescriptions. Table 2 shows the product and prescription counts containing each specified excipient as well as the number of neonates exposed to each PHE. Among PHE-containing products, 29 (55.8%) were parenteral products, 19 (36.5%) were enteral products, and 4 (7.7%) were topical products. Among PHE-containing prescriptions, 284 (44.0%) were parenteral, 308 (47.7%) were enteral, and 54 (8.4%) were topical. When categorized by the administration route, 157 (55.7%), 172 (61.0%), and 57 (20.2%) neonates were exposed to at least one PHE via parenteral, enteral, and topical administration, respectively.

**Table 2:** Number of neonates exposed to potentially hazardous excipients (PHEs) and prescriptions and products containing PHEs

Excipients	PMA at dose (weeks), median (range)	Weight at dose (g), median (range)	Total			Parenteral			Enteral				Topical	
			No. of neonates (***N*** = 343)	No. of prescriptions (***N*** = 2360)	No. of products (***N*** = 426)	No. of neonates (***N*** = 225)	No. of prescriptions (***N*** = 1475)	No. of products (***N*** = 291)	No. of neonates (***N*** = 200)	No. of prescriptions (***N*** = 763)	No. of products (***N*** = 104)	No. of neonates (***N*** = 82)	No. of prescriptions (***N*** = 122)	No. of products (***N*** = 31)
**Aspartame**	45.4 (35.0–67.6)	3375 (1634–3962)	13	15	6	0	0	0	13	15	6	0	0	0
**Benzalkonium chloride**	41.6 (38.6–44.6)	2818 (263.–3006)	3	6	2	0	0	0	0	0	0	3	6	2
**Benzyl alcohol**	34.9 (23.3–71.1)	1808 (486–6665)	100	137	10	98	137	10	0	0	0	0	0	0
**Ethanol**	36.9 (23.9–71.1)	1820 (575–6665)	130	193	8	10	1	2	130	192	6	0	0	0
**Parabens**	38.0 (24.4–71.1)	2062 (410–7505)	86	107	8	13	5	4	43	53	3	49	49	1
**Polysorbate 80**	34.9 (23.3–71.1)	1792 (487–6665)	106	115	17	91	97	8	10	11	6	6	7	3
**Propylene glycol**	36.6 (23.7–55.7)	2116 (486–4175)	71	71	7	39	41	3	30	30	4	0	0	0
**Saccharin sodium**	35.7 (23.9–63.7)	1768 (575–5162)	67	78	8	0	0	0	67	78	8	0	0	0
**Sodium benzoate**	36.7 (23.9–67.6)	1768 (575–5162)	153	217	9	0	0	0	153	215	9	0	0	0
**Sorbitol**	36.9 (23.9–67.6)	1909 (410–5162)	161	170	6	23	30	3	140	140	3	0	0	0
**At least one PHE**	36.0 (23.3–71.1)	1808 (410–7505)	282	646	52	157	284	29	172	308	19	57	54	4

*PMA* postmenstrual age

Among the selected PHEs, benzyl alcohol was the most common PHE in parenteral prescriptions, found in 10 of 291 parenteral products administered to 98 neonates. Sodium benzoate was the most common PHE in enteral prescriptions, found in 9 of 104 enteral products administered to 153 neonates. The most common PHE in topical prescriptions was parabens, found in 1 of 31 topical products administered to 49 (14.3%) neonates. Only 13 neonates were exposed to aspartame via 6 of 104 enteral products.

### Proportion that each product contributes to the total number of products containing each PHE

The proportion that each product contributes to the total number of products containing each PHE was presented in Table 3.

**Table 3:** The proportion that each product contributes to the total number of products containing each potentially harmful excipient

Excipients	Products name	N of prescription	(%)	N of neonates	(%)
**Aspartame**	**Carbocystein dry syrup**	6	(40.0%)	6	(40.0%)
	**Others**	9	(60.0%)	9	(60.0%)
**Ethanol**	**Soluble ferric pyrophosphate syrup**	109	(56.5%)	109	(83.8%)
	**Alfacalcidol solution**	66	(34.1%)	64	(49.2%)
	**Triclofos sodium oral syrup**	14	(7.3%)	14	(10.8%)
	**Others**	4	(2.1%)	4	(3.1%)
**Benzalkonium chloride**	**Atropine ophthalmic solution**	2	(33.3%)	2	(66.7%)
	**Others**	4	(66.7%)	3	(100.0%)
**Benzyl alcohol**	**Heparin sodium injections**	126	(94.2%)	92	(91.1%)
	**Others**	11	(5.8%)	10	(9.9%)
**Parabens**	**Enema solution**	49	(45.8%)	49	(52.7%)
	**Vitamin K2 oral syrup**	28	(26.2%)	28	(30.1%)
	**Amicacin injections**	10	(9.3%)	8	(8.6%)
	**Triclofos sodium oral syrup**	14	(13.1%)	14	(15.1%)
	**Others**	6	(5.6%)	6	(6.5%)
**Polysorbate 80**	**Multivitamin injections**	59	(51.3%)	57	(53.8%)
	**Erythropoietin injections**	37	(32.2%)	35	(33.0%)
	**Others**	19	(16.5%)	17	(16.0%)
**Propylene glycol**	**Multivitamin injections**	40	(56.3%)	38	(53.5%)
	**Vitamin K2 oral syrup**	28	(39.4%)	28	(39.4%)
	**Others**	3	(4.2%)	6	(8.5%)
**Saccharin sodium**	**Multivitamin oral powders**	66	(84.6%)	63	(95.5%)
	**Others**	12	(15.4%)	9	(13.6%)
**Sodium benzoate**	**Soluble ferric pyrophosphate syrup**	109	(50.2%)	109	(71.2%)
	**Multivitamin oral powders**	66	(30.4%)	63	(41.1%)
	**Vitamin K2 oral syrup**	28	(12.9%)	28	(18.3%)
	**Others**	14	(6.5%)	10	(6.5%)
**Sorbitol**	**Soluble ferric pyrophosphate syrup**	109	(64.1%)	109	(67.7%)
	**Vitamin K2 oral syrup**	28	(16.5%)	28	(17.4%)
	**Vitamin K2 injection**	26	(15.3%)	26	(16.1%)
	**Others**	7	(4.1%)	7	(4.3%)

Ethanol was used as a solubilizing agent in soluble ferric pyrophosphate oral syrup, alfacalcidol oral solution, and triclofos sodium oral syrup. These products accounted for 109 (56.5%), 66 (34.1%), and 14 (7.3%), respectively, of 193 ethanol-containing prescriptions. The number of neonates exposed to ethanol via these three drugs was 109 (83.8%), 64 (49.2%), and 14 (10.8%), respectively.

The use of heparin injection for anticoagulation was associated with a higher likelihood of benzyl alcohol use, and heparin injections accounted for 126 (94.2%) of 137 benzyl alcohol-containing prescriptions. More than 90% of benzyl alcohol-exposed neonates were prescribed heparin injections.

Several products contained parabens. Enema formulation accounted for the highest proportion (49 [45.8%] of 107) of paraben-containing prescriptions. Vitamin K2 oral syrup was the second most-used paraben-containing product, accounting for 28 (26.2%) of 107 paraben-containing prescriptions. Together, enema solution and vitamin K2 oral syrup accounted for approximately 80% of all paraben-containing prescriptions.

The use of multivitamin injections for vitamin supplementation and erythropoietin injections for neonatal anemia were associated with a higher likelihood of polysorbate 80 use, responsible for 59 (51.3%) and 37 (32.2%) of 115 polysorbate 80-containing prescriptions, respectively. Multivitamins and erythropoietin injections were prescribed to 57 and 35 neonates, accounting for 53.8 and 33.0% of polysorbate 80 exposures, respectively.

Multivitamin injections and vitamin K2 oral syrup were associated with 40 (56.3%) and 28 (39.4%) of 71 propylene glycol-containing prescriptions, respectively. These two products were prescribed to 38 and 28 neonates, accounting for 53.5 and 39.4% of propylene glycol exposures, respectively.

Oral multivitamin supplementation accounted for 66 (84.6%) of 78 saccharin sodium-containing prescriptions and 63 (95.5%) neonate saccharin sodium exposures.

Most sodium benzoate exposures were due to oral soluble ferric pyrophosphate syrup and multivitamin oral powder administrations, which accounted for 109 (50.2%) and 66 (30.4%) of 217 sodium benzoate-containing prescriptions, respectively. Of 153 sodium benzoate-exposed neonates, 109 (71.2%) and 63 (41.1%) received soluble ferric pyrophosphate syrup and multivitamin oral powder, respectively.

The use of soluble ferric pyrophosphate and vitamin K2 treatments were also associated with a higher likelihood of sorbitol use. These two products were prescribed to 109 (67.7%) and 54 (33.5%) neonates, accounting for 109 (64.1%) and 54 (31.8%) of 170 sorbitol-containing prescriptions. Sorbitol was used as a sweetening agent, diluent for enteral forms, and plasticizer for parenteral forms.

Six of 15 aspartame exposures were due to oral carbocysteine dry syrup, and a small number of neonates were exposed to aspartame.

### Quantitative daily exposure analysis

Quantitative daily exposure was calculated from the package insert or Interview Form information if the quantitative information for each PHE was available. Table 4 shows the number of neonates, PMA (weeks), bodyweight at dose (g), daily dose (mg/kg body weight per day) for each PHE. The numbers of products, neonates, and prescriptions that had no quantitative information were also counted and described. Quantitative PHE information for safety assessments was available only for parenteral products.

**Table 4:** Quantitative analysis of potentially harmful excipient exposure

Excipients	Number of neonates quantitative data available	PMA at dose (weeks)	Weight at dose (g)	Daily PHE Dose (mg/kg/day)	Number of neonates exceed ADI (%)^a^	Missing of quantitative information			ADI for human	Reference no.
		Median (range)	Median (range)	Median (range)		Products	Neonates	Prescriptions		
**Aspartame**	0	–	–	–	–	5	13	13	FDA: 50 mg/kg/day EMA: 40 mg/kg/day (do not apply to infants below 12 weeks of age)	[24, 25, 27]
**Ethanol**	1	32.4	1290.0	1.6	0	5	130	192	6 mg/kg/day	[15]
**Benzalkonium chloride**	0	–	–	–	–	2	3	3	0.1 mg/kg for food residual	[28]
**Benzyl alcohol**	100	34.9 (23.3–71.1)	1808 (486–6665)	0.3 (0.0–48.8)	10 (10%)	0	0	0	5 mg/kg	[29, 30]
**Parabens**	13	30.4 (24.4–42.0)	1101 (410–3102)	0.03 (0.02–0.50)	0 (0%)	4	81	91	10 mg/kg/day	[31]
**Polysorbate 80**	90	34.0 (23.3–55.9)	1646 (487–4175)	1.2 (0.006–31.1)	38 (42.2%)	5	11	11	2 mg (< 1 month) 2–6 mg (< 6 months)	[32]
**Propylene glycol**	39	35.3 (23.7–55.7)	1984 (486–4175)	46.0 (0.04–257.4)	37 (94.9%)	2	28	28	34 mg/kg/day 1 mg/kg/day (< 1 month) 1–50 mg/kg/day (> 1 month)	[33, 34]
**Saccharin sodium**	0	–	–	–	–	5	63	70	15 mg/kg/day	[31]
**Sodium benzoate**	0	–	–	–	–	6	153	208	5 mg/kg/day	[29]
**Sorbitol**	23	35.0 (24.4–40.6)	2098 (410–3716)	8.4 (0.2–13.9)	9 (39.1%)	2	159	136	10 mg/kg/day	[35]

^a^number of neonates exceeding ADI criteria for humans; *PHE* potentially harmful excipient, *PMA* postmenstrual age, *ADI* acceptable daily intake, *FDA* U.S. Food and Drug Administration, *EMA* European Medicines Agency

For the PHEs with available quantitative information, the median daily exposure did not exceed the ADI, except for propylene glycol. The number of neonates that exceeded the reported ADI for each PHE was 10 (10%), 0 (0%), 38 (42.2%), 37 (94.9%), and 9 (39.1%) for benzyl alcohol, parabens, polysorbate 80, propylene glycol, and sorbitol, respectively. Among the paraben products with quantitative data, no cases exceeded the ADI. However, due to the lack of quantitative information for all enteral and topical products, accurate daily PHE exposures could not be determined.

## Discussion

This is the first multicenter prospective observational study examining the administration of PHEs to neonates in Japanese NICUs. While only 12% of the prescribed products contained at least one PHE, 82% of neonates received at least one PHE-containing product. These findings are consistent with previous reports in Estonia, Brazil, and pan-European countries [7, 8, 21].

The descriptive analysis revealed that the administration of heparin for anticoagulation, multivitamins for intravenous hyperalimentation, and erythropoietin injections for anemia in preterm neonates accounted for the majority of PHE-containing prescriptions. The use of heparin injections resulted in frequent benzyl alcohol administration. Similarly, multivitamins, alfacalcidol (vitamin D) oral solution, and soluble ferric pyrophosphate syrup were administered as supplements for vitamin and iron deficiency, which contained polysorbate 80, propylene glycol, ethanol, parabens, benzoates, saccharin sodium, and sorbitol. Erythropoietin injections were also responsible for frequent neonate exposure to polysorbate 80. Among the topical PHE-containing formulations, enema treatments accounted for approximately half of all neonate exposures to parabens. These findings suggest that the use of common and limited medicines was responsible for a large portion of PHE exposure. Replacing these medicines with formulations that do not contain PHEs could significantly reduce neonate PHE exposure.

The quantitative analysis results used to assess the safety of PHE administration in neonates revealed that the median daily exposure to benzyl alcohol, parabens, polysorbate 80, and sorbitol did not exceed the recommended ADI. However, 10–94.9% of neonates received PHE doses exceeding ADIs. Notably, 37 neonates, accounting for 94.9% of all propylene glycol exposures, received doses exceeding the ADI. These neonates received propylene glycol in a multivitamin injection. In addition, a high proportion of sorbitol doses exceeded the ADI. Approximately half of all neonates exposed to polysorbate 80 or sorbitol-containing products received doses exceeding ADIs as a result of multivitamin or vitamin K2 injections. Moreover, quantitative data for enteral and topical products were not available, and accurate daily PHE exposures could not be determined. Benzyl alcohol was the only PHE available with quantitative data available for all benzyl alcohol-containing products, and 10% of neonates exceeded the reported ADI were recorded.

The number of neonates exposed to aspartame was small. Aspartame, is an artificial sweetener, has a potential risk of adverse effects on development in humans with phenylketonuria patients [25]. Because aspartame is frequently used in liquid, syrup, and dry syrup forms, further information on aspartame exposure to infants’ and toddlers’ populations will be required.

When compared our results with the pan-European study [21], the proportion of products containing PHEs was lower in Japan compared with pan-European countries (12.2% of products containing at least one PHE in this study while 26.8% of products contained in the pan-European study), even though the total number of products and prescriptions were similar in the two regions (2360 prescriptions for 426 products in this survey while 2095 prescriptions for 530 products in the pan-European study). Among eight excipients, the proportion of products containing parabens (1.9% vs. 13.4%), propylene glycol (1.6% vs. 4.9%), saccharin sodium (1.9% vs. 5.8%), and sorbitol (1.4% vs. 4.5%) was lower in Japan. Among all prescriptions, the proportion of prescriptions containing PHEs was similar between the two regions (27.4% vs. 30.5%), however, the proportion of prescriptions containing benzalkonium chloride (0.3% vs. 1.3%), parabens (4.5% vs. 18.9%), polysorbate 80 (4.9% vs. 6.6%), propylene glycol (3.0% vs. 6.2%), and saccharin sodium (3.3% vs. 5.0%) was lower in Japan than pan-European countries. Conversely, the proportion of prescriptions containing benzoates (15.0% vs. 4.4%), ethanol (8.2% vs. 2.2%), and sorbitol (7.2% vs. 3.1%) was higher in Japan than pan-European countries. When comparing the proportion of neonates exposed to PHEs, a higher percentage of neonates in NICUs were exposed to PHEs in Japan than pan-European countries (82.2% vs. 62.8%). In particular, the proportion of neonates exposed to ethanol (37.9% vs. 6.5%), benzoates (66.8% vs. 11.3%), polysorbate 80 (30.9% vs. 19.0%), propylene glycol (20.7% vs. 16.5%), saccharin sodium (19.5% vs. 12.4%), and sorbitol (46.9% vs. 7.9%) was higher in Japan. Since the data from the two regions were simply compared and the statistical analysis was not performed, further cross-regional comparison study would be needed. When compared the demographic background of neonates in the pan-European study, median birth weight was smaller (1470 g vs. 1993 g), and the median gestational week was shorter (31.9 weeks vs. 34.0 weeks). These different demographic backgrounds may also reflect the clinical practice and the difference in PHEs exposure between the two regions. Additionally, most of all prescriptions containing PHEs in Japan were derived from nutrition treatment such as iron and vitamins. The current clinical medication practices for neonates in NICUs in Japan may result in frequent PHEs exposure. To reduce PHE exposure in Japan, substitution of the products with PHE-free alternatives will be helpful. For example, a preservative-free heparin injection is now available that does not contain benzyl alcohol; replacement with this PHE-free alternative could theoretically reduce neonate benzyl alcohol exposure by more than 90%. Furthermore, PHE-free oral solid dosage alternatives to alfacalcidol solution and soluble ferric pyrophosphate oral syrup have been developed, which accounted for 96.6% of ethanol exposures in this study.

In contrast to Japan, European Medicines Agency (EMA) proposed several guidances on drug development for the pediatric population. EMA clearly states that constituents of the formulations other than the active substance(s) should be chosen appropriately, avoiding any excipients that are potentially toxic or unsuitable for children [36]. Some aspects of the choice of excipients in respect to carcinogenicity, genotoxicity, and toxicity for reproduction were reviewed by the Committee for Medicinal Products for Human Use. The review includes excipients used in medicinal products intended for children [37]. This stated policy is also adapted in the Paediatric Investigation Plans (PIPs) decision. Although the national regulation on excipients labeling is the same between EMA and Pharmaceuticals and Medical Devices Agency (PMDA) and both two authorities obligate to contain all ingredients used as excipients must be included in the package insert, the scope of regulation seems to be different. The pan-European study was conducted in 2012, and the trends in the use of PHEs in the pediatric formulation may not reflect the current EMA concept adopted in 2013, however, the pediatric formulation will be getting better in European countries in the last 7 years. To clearly describe the difference of excipients’ exposure to neonates in the current situation between Japan and European countries, investigation using the latest information will be also required.

This study has some limitations. First, although the equivalent number of prescription data with the pan-European study was collected in this survey, only 22 (6.2%) NICUs participated in this survey. Second, excipient quantitative data availability was limited. Accurate data on the PHE content in each product are required to evaluate the quantitative neonate PHE exposure. Toxicokinetic studies of PHEs are also recommended because excipients can rapidly reach a toxic level in neonatal populations [38, 39]. Third, this study collected the prescription data for 1 day chosen according to the previous report. However, neonates receiving several medications over a long period may be at risk of cumulative PHE exposure [14, 15]. Longer NICU admission periods are more likely in preterm neonates with poor metabolism and elimination functions [11].

## Conclusion

Neonates admitted in NICUs in perinatal medical centers in Japan were exposed to a number of excipients that are considered to be harmful. The current medication practices in Japanese NICUs result in frequent neonate PHE exposure. Comprehensive benefit and risk assessments of the use of excipients cannot be implemented until all required quantitative data become available. The current PHE administration status and developmental risks warrant further toxicokinetic studies as well as the exploration of feasible alternatives.

## Data Availability

None.

## Abbreviations

PHE: Potentially harmful excipient
NICU: Neonatal intensive care unit
ADI: Acceptable daily intake
API: Active pharmaceutical ingredient
PMA: Postmenstrual age
FDA: U.S. Food and Drug Administration
EMA: European Medicines Agency
PMDA: Pharmaceuticals and Medical Devices Agency
